# The amniotic fluid proteome changes with gestational age in normal pregnancy: a cross-sectional study

**DOI:** 10.1038/s41598-021-04050-9

**Published:** 2022-01-12

**Authors:** Gaurav Bhatti, Roberto Romero, Nardhy Gomez-Lopez, Tinnakorn Chaiworapongsa, Eunjung Jung, Francesca Gotsch, Roger Pique-Regi, Percy Pacora, Chaur-Dong Hsu, Mahendra Kavdia, Adi L. Tarca

**Affiliations:** 1 Perinatology Research Branch, US Department of Health and Human Services, Eunice Kennedy Shriver National Institute of Child Health and Human Development, National Institutes of Health, Detroit, MI USA; 2 Department of Obstetrics and Gynecology, Wayne State University School of Medicine, Detroit, MI USA; 3 Department of Biomedical Engineering, Wayne State University College of Engineering, Detroit, MI USA; 4 Department of Obstetrics and Gynecology, University of Michigan, Ann Arbor, MI USA; 5 Department of Epidemiology and Biostatistics, Michigan State University, East Lansing, MI USA; 6 Center for Molecular Medicine and Genetics, Wayne State University, Detroit, MI USA; 7 Detroit Medical Center, Detroit, MI USA; 8 Department of Biochemistry, Microbiology and Immunology, Wayne State University School of Medicine, Detroit, MI USA; 9 Office of Women’s Health, Integrative Biosciences Center, Wayne State University, Detroit, MI USA; 10 Department of Physiology, Wayne State University School of Medicine, Detroit, MI USA; 11 Department of Computer Science, Wayne State University College of Engineering, Detroit, MI USA; 12 Present Address: Department of Obstetrics, Gynecology & Reproductive Sciences, The University of Texas Health Sciences Center at Houston, Houston, TX USA; 13 Present Address: Department of Obstetrics & Gynecology, University of Arizona College of Medicine -Tucson, Tucson, AZ USA

**Keywords:** Intrauterine growth, Intrauterine growth, Diagnostic markers, Proteome informatics, Gene expression analysis, Proteomic analysis, Proteomics

## Abstract

The cell-free transcriptome in amniotic fluid (AF) has been shown to be informative of physiologic and pathologic processes in pregnancy; however, the change in AF proteome with gestational age has mostly been studied by targeted approaches. The objective of this study was to describe the gestational age-dependent changes in the AF proteome during normal pregnancy by using an omics platform. The abundance of 1310 proteins was measured on a high-throughput aptamer-based proteomics platform in AF samples collected from women during midtrimester (16–24 weeks of gestation, n = 15) and at term without labor (37–42 weeks of gestation, n = 13). Only pregnancies without obstetrical complications were included in the study. Almost 25% (320) of AF proteins significantly changed in abundance between the midtrimester and term gestation. Of these, 154 (48.1%) proteins increased, and 166 (51.9%) decreased in abundance at term compared to midtrimester. Tissue-specific signatures of the trachea, salivary glands, brain regions, and immune system were increased while those of the gestational tissues (uterus, placenta, and ovary), cardiac myocytes, and fetal liver were decreased at term compared to midtrimester. The changes in AF protein abundance were correlated with those previously reported in the cell-free AF transcriptome. Intersecting gestational age-modulated AF proteins and their corresponding mRNAs previously reported in the maternal blood identified neutrophil-related protein/mRNA pairs that were modulated in the same direction. The first study to utilize an aptamer-based assay to profile the AF proteome modulation with gestational age, it reveals that almost one-quarter of the proteins are modulated as gestation advances, which is more than twice the fraction of altered plasma proteins (~ 10%). The results reported herein have implications for future studies focused on discovering biomarkers to predict, monitor, and diagnose obstetrical diseases.

## Introduction

Amniotic fluid (AF) provides nutrition, physical protection, and antimicrobial defenses to the fetus^1^. The volume and composition of AF change with gestational age^1,2^. Early in pregnancy, there is a relatively free exchange of water, nutrients, and molecules across the fetal skin and chorioamniotic membranes into the amniotic cavity^2,3^. Thus, the AF composition is similar to maternal and fetal plasma during this period^2,3^. However, as the fetal skin keratinizes between 22 and 25 weeks of gestation, fetal secretions, especially urine, become the most significant contributors to AF composition^2,3^. Subsequently, throughout mid and late pregnancy, AF contains biological signals such as proteins, nucleic acids, and metabolites that can provide a unique window into fetal well-being^4^.

The AF proteome not only provides information about fetal genotype and growth but also reflects the adaptations of maternal–fetal physiology during the progression of pregnancy^5,6^. Disruption of these tightly regulated maternal–fetal interactions underlies the complications associated with pregnancy, which include the “great obstetrical syndromes”^5–7^. Both fetal and maternal tissues contribute to the AF proteome, making it a reservoir of potential protein biomarkers that allow monitoring of fetal health and detect developing pathologies^6,8^. Moreover, the comparisons between proteomic profiles from diseased and healthy pregnancies may also elucidate the etiologies of obstetrical syndromes^5^. Indeed, several studies have examined the AF proteome in fetal genetic disorders, such as trisomy 21 (i.e., Down syndrome)^9^ and Turner syndrome^10^, as well as in pregnancy complications that may include polyhydramnios^11^, intra-amniotic inflammation^12^, preterm prelabor rupture of the membranes^13^, preeclampsia^14^, and spontaneous preterm labor^15^. Consequently, several AF proteins have been proposed as biomarkers for perinatal complications^3^.

One of the most prominent AF proteins in the context of intra-amniotic inflammation, intra-amniotic infection, and spontaneous preterm delivery is interleukin (IL)-6^16–21^. A rapid point-of-care diagnostic test based on AF IL-6 concentrations showed predictive value for intra-amniotic inflammation (sensitivity = 93%, specificity = 91%) and microbial invasion of the amniotic cavity (sensitivity = 91%, specificity = 62%)^20^. Moreover, second-trimester AF concentrations of α-fetoprotein and acetylcholinesterase have been used to detect fetal neural tube defects such as spina bifida^22^. However, few AF proteins have shown widespread utility for routine clinical practice, given that amniocentesis is an invasive, high-risk procedure typically performed only once between 15 and 20 weeks of gestation^23^. Thus, the focus has been shifted to minimally invasive prenatal diagnostics^24^ based on ultrasound and testing of maternal fluids such as the peripheral blood^25^, saliva^26^, and urine^27^. Nevertheless, given the proximity of AF to the gestational tissues and the abundance of fetus-derived proteins^5,28^ in this compartment, AF remains the preferred choice for unbiased studies exploring the pathology of obstetrical diseases^3,29^. Once the underlying disease pathways have been determined, related biomarkers can be targeted in subsequent hypothesis-driven studies to measure the more easily accessible body fluids^30^.

The AF proteome must be evaluated in healthy term pregnancies to establish the AF physiologic composition prior to investigating pregnancy-related pathological conditions^31^. This “normal” AF proteome could then serve as a reference to determine whether the profiles of specific proteins are perturbed in obstetrical diseases^31,32^. Several groups have characterized the normal AF proteome by mass spectrometry (MS), which has been the preferred method since 2004^3,5,31,33–35^. Most of these studies have examined second-trimester samples when amniocentesis is most commonly performed; yet, few have compared the AF proteome between different trimesters of pregnancy^36,37^. Moreover, variations in protocols for sample preparation, protein separation, depletion of high-abundance proteins, and analysis methods have made it difficult to accurately compare prior studies^38^. Thus, there remains a need for a more precise high-throughput quantification of gestational age-dependent changes in the AF proteome.

Herein, we have applied an aptamer-based proteomics platform to assess the effects of gestational age on the AF proteome in normal pregnancy^39,40^. The assay can simultaneously measure thousands of proteins with high sensitivity and dynamic range^41^. This platform has been previously applied to describe gestational age-dependent changes in the maternal plasma^32^ and to identify candidate biomarkers of preeclampsia^42–44^ and the spectrum of placenta accreta^45^. SomaSignal tests based on reproducible proteomic signals derived from SOMAscan assays have been shown to provide actionable, personalized clinical information for multiple human diseases and conditions^46–48^.

## Results

### Demographic characteristics of the study population

**Figure 1:**
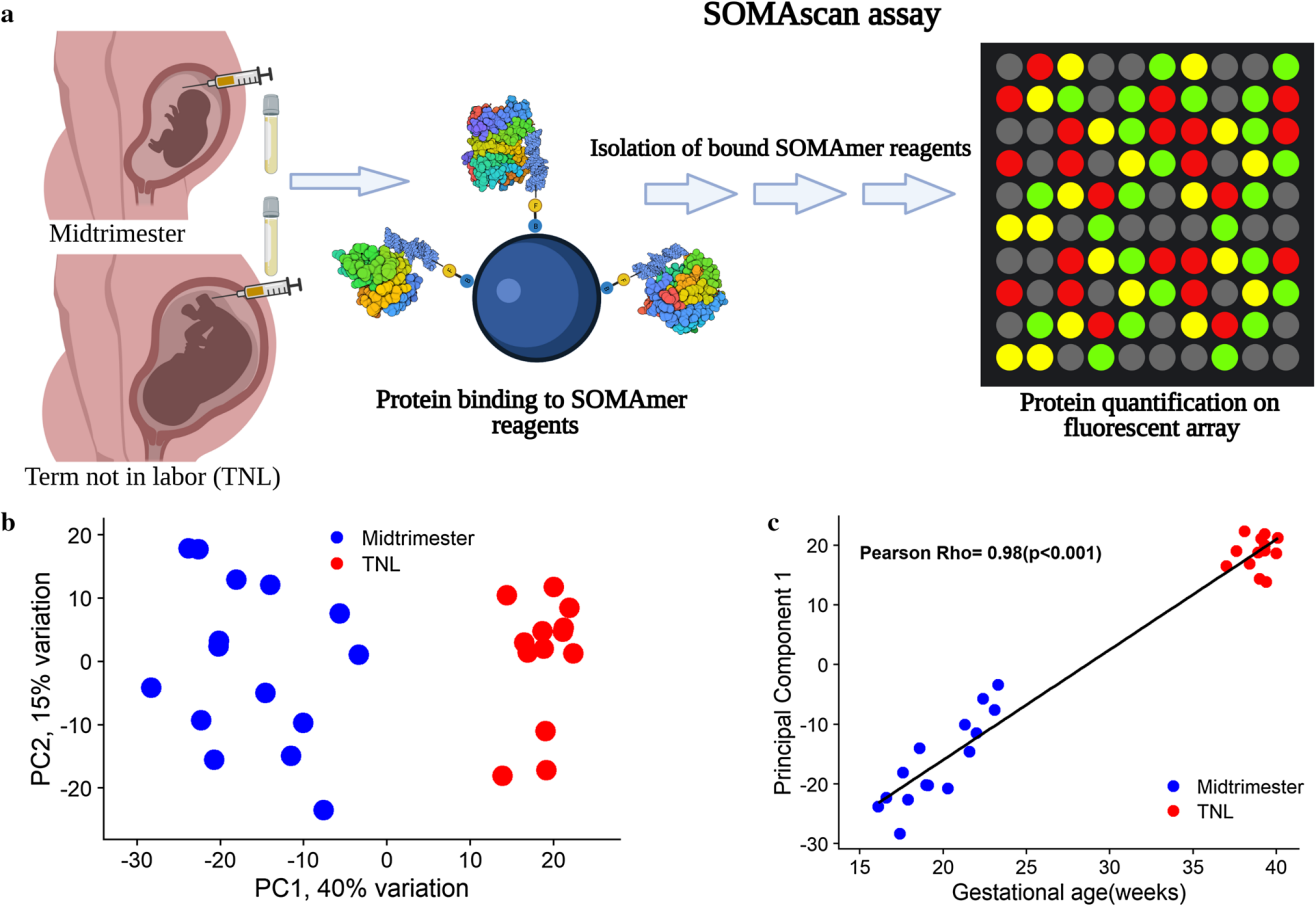
Gestational age-dependent changes in the amniotic fluid proteome. (**a**) The abundance of 1310 proteins was determined by the SOMAmer (Slow Off-rate Modified Aptamers) platform in amniotic fluid samples collected from pregnant women during midtrimester (16–24 weeks, n = 16) and from pregnant women at term without labor (37–42 weeks, n = 14). The figure was created with biorender.com based on the description of SOMAscan assay^101^. (**b**) Principal component analysis showing all samples depicted as their first and second principal components derived from the amniotic fluid proteomic data. The proportion of variance explained by each principal component is shown along the axis. The R package, *PCAtools*, was used to calculate and plot the principal components^114^. (**c**) Scatter plot (created in R^115^) shows the linear correlation between gestational age and the first principal component along with the Pearson correlation coefficient.

The abundance of 1310 proteins was profiled (Fig. 1a) in AF samples collected from pregnant women during midtrimester (n = 15) and pregnant women at term without labor (TNL, n = 13). The comparison of clinical characteristics between the two groups is shown in Table 1. The median gestational age at the time of sample collection was 19.2 weeks and 39 weeks in the midtrimester and TNL groups, respectively. Women in the midtrimester group were older (median maternal age: 30 years vs. 22 years, *p* = 0.012) and bore more female fetuses (60% vs. 15.4%, p = 0.024) than those in the TNL group. There was also a significant difference in the rate of cesarean delivery between groups (*p* = 0.006, Table 1).

**Table 1:** Demographic characteristics of the women included in the proteomics study. Continuous variables were compared with a Welch’s t-test and are summarized as medians (interquartile range). Categorical variables are shown as number (%) and were compared by using Fisher’s exact test.

	TNL (n = 13)	Midtrimester (n = 15)	*p*-value
Age (years)	22 (21–29)	30 (26–32)	0.012
BMI*(kg/m^2^)	33.6 (30.8–35.4)	28.3 (25.9–29.8)	0.151
Pre-pregnancy weight (lbs.)	194.5 (170.5–200.2)	168 (147.5–199.5)	0.354
Height (cm)	162.6 (157.5–167.6)	165.1 (156.2–172.7)	0.639
African-American race (%)	10/13 (76.9%)	13/15 (86.7%)	0.639
Smoking status (%)	3/13 (23.1%)	1/15 (6.7%)	0.311
Alcohol use (%)	0/13 (0%)	0/15 (0%)	1
Nulliparity (%)	2/13 (15.4%)	2/15 (13.3%)	1
History of preterm birth (%)	2/13 (15.4%)	0/15 (0%)	0.206
Gestational age at amniocentesis (weeks)	39.1 (38.4–39.3)	19.1 (17.8–21.8)	< 0.001
Gestational age at delivery (weeks)	39.1 (38.4–39.4)	39 (38.5–40.1)	0.557
Spontaneous labor (%)	0/13 (0%)	11/15 (73.3%)	< 0.001
Cesarean delivery (%)	12/13 (92.3%)	6/15 (40%)	0.006
Female fetus (%)	2/13 (15.4%)	9/15 (60%)	0.024
Birthweight (g)	3475 (3210–3675)	3185 (3092.5–3350)	0.077

*BMI* body mass index; TNL: term not in labor.

*Contains one missing data.

### Effect of gestational age on the AF proteome

Unsupervised data representation of the AF proteome with principal components analysis (Fig. 1b) showed a clear separation between the midtrimester and TNL samples. The first principal component captured 40% of the variation in the protein abundance and was linearly correlated with gestational age at sampling (Pearson correlation = 0.98, *p* < 0.001, Fig. 1c).

#### Differential protein abundance

**Figure 2:**
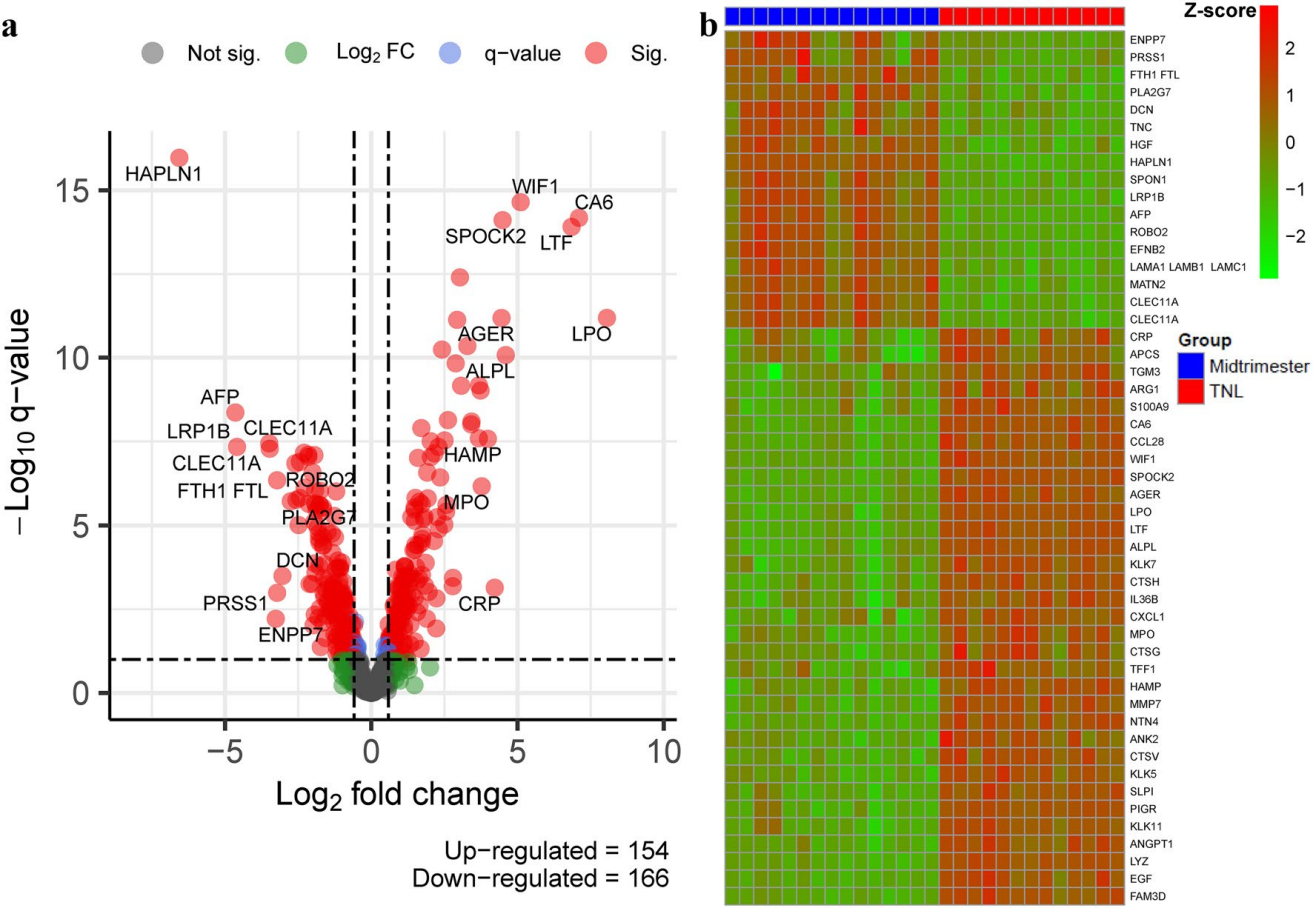
Differential protein abundance. The figure shows (**a**) the volcano plot of log_10_ transformed adjusted  *p*-values against log_2_ transformed fold changes of 1310 amniotic fluid proteins and (**b**) the heatmap based on the 50 most-increased (in abundance) and the 50 most-decreased (in abundance) proteins between term and midtrimester samples. The R/Bioconductor packages, *EnhancedVolcano*, and *pheatmap*, were used to generate the volcano plot and heatmap, respectively^115–117^.

A comparison of 1310 AF proteins between the midtrimester and TNL groups, while adjusting for fetal sex and maternal age, identified 320 proteins modulated with advancing gestational age in normal pregnancy (q-value < 0.1 and fold change ≥ 1.5) (Table S1). Of these, 51.9% (166/320) showed lower abundance, and 48.1% (154/320) were increased at term compared to midtrimester (Fig. 2a). Table 2 lists the 100 most highly modulated proteins (based on fold change), and Fig. 2b shows the expression profile of these proteins across all AF samples. The log_2_ fold changes between term and midtrimester groups for these 320 proteins were highly consistent regardless of the adjustment for BMI (correlation coefficient 0.998 and a 100% agreement in the direction of change between groups). Thus, the protein dysregulation that we observed could not be attributed to differences in BMI between the groups.

**Table 2:** List of the top 100 amniotic fluid proteins that significantly change in abundance between gestational-age groups in normal pregnancy. The table consists of the gene symbol, protein name, direction of change, and fold change (Term not in labor vs. Midtrimester).

SYMBOL	Name	Direction	Fold
LPO	Lactoperoxidase	Up	266
CA6	Carbonic anhydrase 6	Up	137
LTF	Lactotransferrin	Up	115
WIF1	Wnt inhibitory factor 1	Up	34.4
ALPL	Alkaline phosphatase, tissue-nonspecific isozyme	Up	24.2
SPOCK2	Testican-2	Up	22.4
AGER	Advanced glycosylation end product-specific receptor, soluble	Up	21.9
CRP	C-reactive protein	Up	18.6
HAMP	Hepcidin	Up	15.8
MPO	Myeloperoxidase	Up	13.7
MMP7	Matrilysin	Up	13.3
SLPI	Antileukoproteinase	Up	12.8
KLK7	Kallikrein-7	Up	12.8
PIGR	Polymeric immunoglobulin receptor	Up	10.7
CTSH	Cathepsin H	Up	10.7
CCL28	C–C motif chemokine 28	Up	9.7
EGF	Epidermal growth factor	Up	8.4
LYZ	Lysozyme C	Up	8.2
NTN4	Netrin-4	Up	7.6
CTSV	Cathepsin L2	Up	7.4
TGM3	Protein-glutamine gamma-glutamyltransferase E	Up	6.9
TFF1	Trefoil factor 1	Up	6.9
KLK5	Kallikrein-5	Up	6.1
ANK2	Ankyrin-2	Up	5.9
CXCL1	Growth-regulated alpha protein	Up	5.9
KLK11	Kallikrein-11	Up	5.7
ARG1	Arginase-1	Up	5.6
FAM3D	Protein FAM3D	Up	5.3
S100A9	Protein S100-A9	Up	5.1
IL36B	Interleukin-36 beta	Up	5
ANGPT1	Angiopoietin-1	Up	4.9
CTSG	Cathepsin G	Up	4.8
APCS	Serum amyloid P-component	Up	4.7
SERPINE1	Plasminogen activator inhibitor 1	Up	4.7
CTSS	Cathepsin S	Up	4.5
BPI	Bactericidal permeability-increasing protein	Up	4.4
IL1RN	Interleukin-1 receptor antagonist protein	Up	4.1
MED1	Mediator of RNA polymerase II transcription subunit 1	Up	4.1
COLEC12	Collectin-12	Up	3.9
CXCL6	C-X-C motif chemokine 6	Up	3.8
MIA	Melanoma-derived growth regulatory protein	Up	3.8
CXCL8	Interleukin-8	Up	3.7
PDIA3	Protein disulfide-isomerase A3	Up	3.6
PGLYRP1	Peptidoglycan recognition protein 1	Up	3.6
SCARB2	Lysosome membrane protein 2	Up	3.5
GNS	N-acetylglucosamine-6-sulfatase	Up	3.5
ESM1	Endothelial cell-specific molecule 1	Up	3.4
TKT	Transketolase	Up	3.3
CHRDL1	Chordin-like protein 1	Up	3.3
KLK8	Kallikrein-8	Up	3.3
HAPLN1	Hyaluronan and proteoglycan link protein 1	Down	94.7
AFP	alpha-Fetoprotein	Down	25.1
LRP1B	Low-density lipoprotein receptor-related protein 1B	Down	24.1
CLEC11A	Stem cell growth factor-alpha	Down	11.2
ENPP7	Ectonucleotide pyrophosphatase/phosphodiesterase family member 7	Down	9.6
FTH1 FTL	Ferritin	Down	9.3
PRSS1	Trypsin-1	Down	9.3
DCN	Decorin	Down	8.2
PLA2G7	Platelet-activating factor acetylhydrolase	Down	6.8
ROBO2	Roundabout homolog 2	Down	6.1
SPON1	Spondin-1	Down	5.9
TNC	Tenascin	Down	5.6
EFNB2	Ephrin-B2	Down	5.5
LAMA1 LAMB1 LAMC1	Laminin	Down	5.4
MATN2	Matrilin-2	Down	4.9
HGF	Hepatocyte growth factor	Down	4.9
SERPINA1	Alpha-1-antitrypsin	Down	4.5
DKK3	Dickkopf-related protein 3	Down	4.4
TGFBI	Transforming growth factor-beta-induced protein ig-h3	Down	4.4
PGAM1	Phosphoglycerate mutase 1	Down	4.3
C1QA C1QB C1QC	Complement C1q subcomponent	Down	4.1
NOTCH3	Neurogenic locus notch homolog protein 3	Down	4
PRSS2	Trypsin-2	Down	3.9
CD163	Scavenger receptor cysteine-rich type 1 protein M130	Down	3.9
LSAMP	Limbic system-associated membrane protein	Down	3.9
CGA CGB	Human Chorionic Gonadotropin	Down	3.8
IGFBP1	Insulin-like growth factor-binding protein 1	Down	3.8
LTBP4	Latent-transforming growth factor beta-binding protein 4	Down	3.8
EPHA5	Ephrin type-A receptor 5	Down	3.8
APOE	Apolipoprotein E (isoform E3)	Down	3.8
MRC2	C-type mannose receptor 2	Down	3.7
F10	Coagulation factor Xa	Down	3.7
TGFBR3	Transforming growth factor beta receptor type 3	Down	3.6
ANP32B	Acidic leucine-rich nuclear phosphoprotein 32 family member B	Down	3.5
UNC5D	Netrin receptor UNC5D	Down	3.5
SERPING1	Plasma protease C1 inhibitor	Down	3.4
MFGE8	Lactadherin	Down	3.4
TNFRSF21	Tumor necrosis factor receptor superfamily member 21	Down	3.4
SPARC	SPARC	Down	3.4
ACY1	Aminoacylase-1	Down	3.4
ECM1	Extracellular matrix protein 1	Down	3.4
CGA LHB	Luteinizing hormone	Down	3.3
EFNB1	Ephrin-B1	Down	3.2
C1R	Complement C1r subcomponent	Down	3.2
NRP1	Neuropilin-1	Down	3.1
FN1	Fibronectin Fragment 3	Down	3.1
DSC2	Desmocollin-2	Down	3.1
APOB	Apolipoprotein B	Down	3.1
SIGLEC14	Sialic acid-binding Ig-like lectin 14	Down	3.1
NBL1	Neuroblastoma suppressor of tumorigenicity 1	Down	3

**Figure 3:**
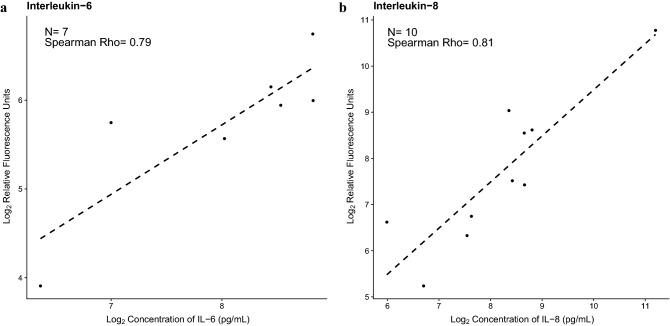
Correlation between SOMAscan assay and the corresponding ELISA assay for differentially abundant proteins. The figure shows the scatter plots (created in R^115^) of log_2_ transformed relative fluorescence units determined with SOMAscan assay on the y- axis and log_2_ transformed ELISA concentrations (pg/ml) on the x-axis for (**a**) interleukin (IL)-6, and (**b**) IL-8. Spearman’s correlation coefficients are also shown.

For a subset of AF samples included in this study, the concentrations of two differentially abundant proteins (IL-6 and IL-8) had been previously determined by ELISA. A Spearman correlation analysis showed a significant positive correlation between proteomic platforms for these proteins, suggesting that the observed changes are reproducible (Rho ≈ 0.8, *p* < 0.05 for both) (Fig. 3a,b).

**Figure 4:**
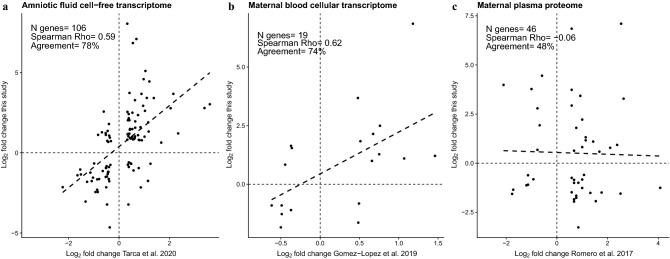
Correlation between gestational age-dependent changes in the amniotic fluid proteome and those previously reported in the cell-free amniotic fluid transcriptome, maternal blood cellular transcriptome, and maternal plasma proteome. Scatter plots (created in R^115^) show the Spearman’s correlation between log_2_ fold changes between the gestational-age groups obtained in this study for amniotic fluid proteins (on the y-axis ) and those previously reported for corresponding proteins or genes (on the x-axis) in (**a**) cell-free amniotic fluid transcriptome, (**b**) maternal blood cellular transcriptome, and (**c**) maternal plasma proteome. For each pairwise comparison, only the molecules (genes/proteins) significant in both studies were included in the correlation analysis.

We then compared the gestational age-dependent changes in AF proteins to previously reported gestational age-dependent changes in the AF cell-free transcriptome^49^, maternal plasma proteome^32^, and maternal blood cellular transcriptome^50^. For the 106 gene/protein pairs measured and found significant herein and by cell-free transcriptome analysis^49^, the log_2_ fold change (term/midtrimester) correlation was significant (Spearman’s correlation = 0.59, *p* < 0.001), with 78% (83/106) of the genes/proteins changing in the same direction between studies (Fig. 4a). Nineteen genes coding for proteins significantly changing with gestational age were found by integrating the maternal blood cellular transcriptome results^50^ and AF proteomics data. The log_2_ fold changes were significantly correlated (Spearman correlation = 0.62, *p* = 0.004) between mRNA/protein pairs, and 74% (14/19) of the genes changed in the same direction as corresponding proteins (Fig. 4b). These 14 genes included lactotransferrin (*LTF*), cysteine rich secretory protein 3 (*CRISP3*), bactericidal permeability increasing protein (*BPI*), oxidized low density lipoprotein receptor 1 (*OLR1*), arginase 1 (*ARG1*), transforming growth factor beta receptor 3 (*TGFBR3*), immunoglobulin heavy constant mu (*IGHM*), peptidoglycan recognition protein 1 (*PGLYRP1*), immunoglobulin heavy constant alpha 1 (*IGHA1*), S100 calcium binding protein A12 (*S100A12*), CD177 molecule (*CD177*), peptidase inhibitor 3 (*PI3*), secretory leukocyte peptidase inhibitor (*SLPI*), and joining chain of multimeric IgA and IgM (*JCHAIN*).

No correlation was observed between gestational age-dependent changes in the AF proteome and maternal plasma proteome (Fig. 4c).

#### Biological processes modulated with advancing gestation in the AF proteome

The five most-increased proteins modulated with gestational age were lactoperoxidase (LPO), carbonic anhydrase 6 (CA6), lactotransferrin (LTF), Wnt inhibitory factor 1 (WIF1), and alkaline phosphatase, tissue-nonspecific isozyme (ALPL). Functional analysis of all up-regulated proteins showed enrichment of 48 biological processes, 12 molecular functions, and 45 cellular components (Table S2). Myeloid leukocyte-mediated immunity, exocytosis, cell redox homeostasis, maternal process involved in female pregnancy, and cellular response to thyroid hormone stimulus were among the significantly enriched biological processes. Enriched cellular components included the extracellular region (extracellular space and extracellular matrix), vesicle, and endomembrane system.

The most decreased proteins at term were hyaluronan and proteoglycan link protein 1 (HAPLN1), α-fetoprotein (AFP), low-density lipoprotein receptor-related protein 1B (LRP1B), ectonucleotide pyrophosphatase/phosphodiesterase family member 7 (ENPP7), and stem cell growth factor-α (CLEC11A). Gene Ontology (GO) enrichment analysis of genes coding for the down-regulated proteins identified three biological processes, 35 cellular components, and 10 molecular functions (Table S3). The over-represented biological processes were related to extracellular organization, and the enriched cellular components included the basement membrane, extracellular region, endoplasmic reticulum lumen, apical junction complex, cell periphery, and membrane. Extracellular matrix structural constituent, calcium ion binding, signaling receptor activity, and molecular transducer activity were among the over-represented molecular functions among the proteins decreasing in abundance with gestational age.

#### AF protein modulation during the midtrimester of pregnancy

In addition to examining changes in the AF proteome between midtrimester and term, we also assessed the changes in AF protein abundance between the early (16.4–21.0 weeks of gestation) and the late (21.1–24.0 weeks of gestation) midtrimesters. We identified 236 differentially abundant proteins between the early and late midtrimesters, of which the large majority (230, 97%) were down-regulated at late midtrimester (Table S4).

#### Clustering of gestational age-modulated proteins

**Figure 5:**
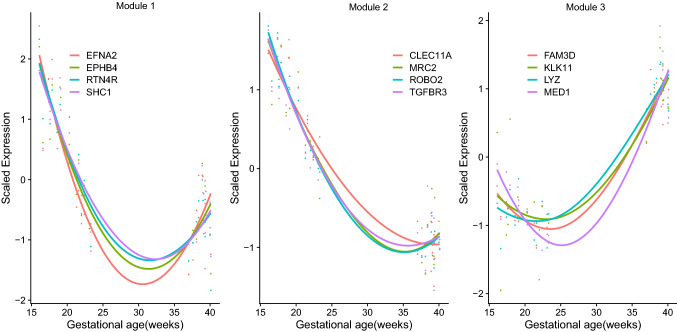
Clustering of amniotic fluid protein profiles during gestation. The figure shows locally estimated scatterplot smoothing (LOESS) regression plots (created in R^115^) of gestational age-dependent abundance profiles of four intra-modular hub proteins in the three modules identified by weighted correlation network analysis (WGCNA).

The comparison between midtrimester and term samples identified 320 differentially abundant proteins, whereas the comparison between early and late midtrimester samples identified 236 differentially abundant proteins. Overall, 429 unique proteins were significantly modulated by gestational age. We carried out a weighted correlation network analysis (WGCNA) of these proteins to identify modules of interconnected proteins. WGCNA identified three protein modules (module 1: n = 131, module 2: n = 121, and module 3: n = 154 member proteins) (Fig. 5). Twenty-three proteins were assigned to the background or noise module. Figure 5 shows the changes in standardized protein expression over gestational age of four intramodular hubs corresponding to each of the three modules. Notably, protein expression decreased monotonically during the midtrimester in modules 1 and 2 before increasing again at term in module 1, but not in module 2. By contrast, module 3 proteins increased from midtrimester to term gestation.

### Changes in tissue-specific signatures with gestational age

**Figure 6:**
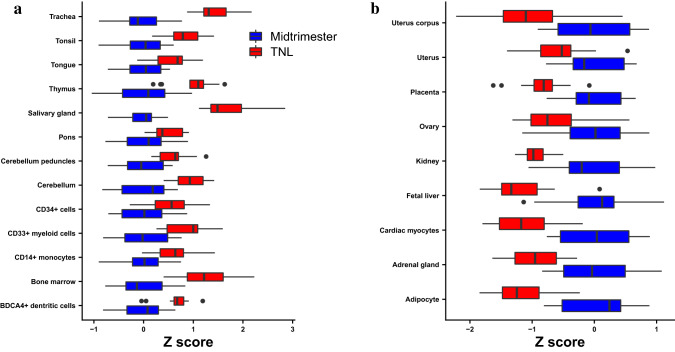
Expression of tissue-specific signatures. For each tissue, the expression of proteins coded by up to the 20 most preferentially expressed genes according to the GNF Gene Expression Atlas was transformed into a Z-score and averaged. The Z-scores were compared between term and midtrimester samples. Tissues with significantly (q-value < 0.05) (**a**) increased and (**b**) decreased expression at term not in labor (TNL) compared to midtrimester are shown.The box plots were created in R^115^.

To gain further biological insight from the proteomic abundance modulation with gestational age, we analyzed the average standardized expression (Z-scores) of proteins coded by tissue-specific genes, defined according to the GNF Gene Expression Atlas^51^. The Z-scores of 13 tissue and cell type-specific signatures were significantly increased at term compared to midtrimester (q < 0.05, Fig. 6a). These included signatures of the respiratory tract (trachea), brain (pons, cerebellum peduncles, and cerebellum), tongue, salivary gland, and immune system (bone marrow, thymus, whole blood, monocytes, and dendritic cells). In addition, the Z-scores of nine tissues/cell types were significantly decreased at term compared to midtrimester (q < 0.05, Fig. 6b), including gene signatures of the kidney, fetal liver, placenta, uterus, and cardiac myocytes.

## Discussion

Herein, we performed the first aptamer-based proteomic profiling of AF and demonstrated that almost 25% (320) of the 1310 AF proteins measured changed in abundance between midtrimester and term pregnancy. This fraction of the AF proteome, modulated by gestational age, is more than twice found in previously reported studies of the maternal plasma proteome, as assessed on an earlier version of the same platform, involving 1125 of the 1310 proteins profiled herein^32^. Specifically, the concentrations of 12 proteins (LPO, CA6, LTF, WIF1, ALPL, SPOCK2, AGER, CRP, HAMP, HAPLN1, AFP, and LRP1B) were changed more than 15-fold at term compared to midtrimester. Our results regarding expected patterns of modulation of the proteome in AF during normal pregnancy can serve as a reference for future studies focused on discovering biomarkers to predict, monitor, and diagnose pregnancy-related diseases^31,32^.

There has been considerable interest in ascertaining the composition and function of the normal AF proteome^3^. An early report utilized mass spectrometry to identify 59 unique proteins in an AF sample collected at 15 weeks of gestation from a 36-year-old healthy mother^33^. A later study described the cellular proteome of AF samples collected between 16 and 18 weeks of gestation^5,34^ and identified 432 different gene products in the AF cellular proteome. The majority were enzymes, structural proteins, and transport proteins^34^. The same group later reported 136 proteins in the soluble fraction of AF, of which only seven were also present in the AF cellular proteome^5^. Two subsequent studies, one examining the second-trimester normal AF proteome with three complementary protein separation techniques^3^ and the other comparing the AF transcriptome between normal and trisomy 21 pregnancies, increased the AF proteins to 965^35^. More recently, the known number of proteins constituting the AF proteome between 16 and 20 weeks of gestation was increased to 2881^31^. Proteomic comparison between five different biological fluids showed that 371 of these 2881 proteins were unique to AF^52^. All of these studies utilized AF samples collected during the second trimester. Furthermore, combining and reproducing the aforementioned MS-based proteomics studies has been challenging given the differences in sample preparation, protein separation, depletion of high-abundance proteins, and bioinformatics approaches, among other factors^38^.

There is a lack of information related to AF proteome dynamics throughout gestation. One report utilized two-dimensional fluorescence difference gel electrophoresis (2D-DIGE), two-dimensional gel electrophoresis, and silver staining to identify differentially expressed protein spots between AF collected during the 17th and 40th weeks of gestation^36^. Another study also utilized 2D-DIGE to compare the AF proteomes among the three trimesters of pregnancy and confirmed that AF protein composition is dependent on gestational age^37^. There remains a need to characterize the gestational age-dependent changes in the normal AF proteome, utilizing a high-throughput assay to simultaneously measure a large number of proteins and to assess common, novel information that is not attainable by other omics approaches (e.g., transcriptomics). Accordingly, the SOMAscan platform used herein is an affinity-based proteomics platform that uses modified aptamers known as SOMAmers to target thousands of proteins in a single run without the need to fractionate the sample^39,40,53^.

Herein, we found that the AF abundance of nine proteins (LPO, CA6, WIF1, ALPL, SPOCK2, AGER, CRP, and HAMP) was significantly increased at term compared to midtrimester. Of note, such a dynamic range of modulation was previously observed for only three proteins in the maternal plasma proteome, namely placental growth factor (PlGF, 14.5-fold), glypical-3 (GPC3, 26-fold), and sialic acid-binding Ig-like lectin 6 (SIGLEC6, 16.9-fold)^32^. LPO protects the fetal airway system against invading pathogens and may also act as an antioxidant by scavenging hydrogen peroxide^54–56^. CA6 is implicated in pH homeostasis^57^, and its concentration in maternal plasma was previously shown to increase with gestation^32^. Other proteins showing dramatic elevation at term compared to midtrimester are secreted by the lung, liver, brain, and retina^58^. WIF1, a Wnt signaling inhibitor, is expressed by the Müller glial cells of the retina and has been linked to multiple functions, including chondrogenesis; eye, lung, and anorectal development; neurogenesis; and tooth morphogenesis^58,59^. ALPL is expressed by many tissues (e.g., lung, liver, and blood) and is implicated in bone and tooth mineralization^58,60^. SPOCK2 is an extracellular chondroitin and heparan sulfate proteoglycan expressed in the brain and lungs^61^; in the latter organ, SPOCK2 is expressed by type 1 Alveolar cells and plays a role in fetal lung development, most likely through interactions with matrix metalloproteinases^58,62^. The elevated concentrations of AGER, CRP, and HAMP at term compared to midtrimester AF samples are consistent with previous reports^63,64^. AGER, a multi-ligand receptor expressed in the lungs^58^, is implicated in multiple biological processes, including homeostasis, development, and inflammation^65^. Other sources of soluble AGER in the amniotic cavity include amnion epithelial cells, extravillous trophoblasts, decidual cells, and neutrophils^63,66^. The AF concentrations of sRAGE significantly increase with gestational age before decreasing during spontaneous labor^63,66^, which may be attributed to preparation for imminent delivery, and the increase in the abundance of its ligands during labor may be responsible for the consumption and subsequent decrease of AF sRAGE during labor^63^. CRP and HAMP, two acute-phase response proteins secreted by the liver, are involved in defense against invading microorganisms^67,68^. The AF CRP concentration is increased in women with preterm labor and intra-amniotic infection^69^. CRP acts as a pattern recognition receptor for pathogen- and damage-associated molecular patterns (PAMPs and DAMPs, respectively) and activates the classical complement pathway, among other immune effector processes^65,70^. Thus, proteins that are highly modulated with advancing gestational age include antimicrobial, developmental, and inflammatory molecules.

Consistent with the above observations, GO analysis of AF proteins up-regulated at term compared to midtrimester samples identified terms related to immune effector processes involved in defense against invading microbes. In particular, terms related to neutrophil-mediated immunity were the most common among enriched gene ontologies, which aligns with a previous report showing that neutrophils represent the dominant immune cell subset in amniotic fluid during term gestation^71^. Moreover, neutrophils in the amniotic cavity are functional and capable of carrying out multiple host-defense mechanisms, including the formation of neutrophil extracellular traps, the performance of phagocytosis, and the release of cytokines and antimicrobial products^72–78^.

The most down-regulated AF proteins at term compared to midtrimester were HAPLN1, AFP, and LRP1B. HAPLN1 stabilizes the interactions between hyaluronic acid and proteoglycans, such as aggrecan and versican, in the extracellular matrix^79–81^, and the biological functions of HAPLN1 during fetal growth include chondrocyte differentiation and cartilage development^82^, heart development^83^, neural differentiation^84^, and neocortical folding ^85^. LRP1B is a cell-surface receptor involved in receptor-mediated endocytosis^86^, expressed in the fetal brain^87^, where it may regulate apolipoprotein-mediated cholesterol uptake^88^. AFP is produced by the fetal liver^51,65^, and amniotic fluid concentrations of AFP are clinically utilized to screen for birth defects and genetic abnormalities^22^. AFP modulates sexual differentiation in the fetal brain by binding to estrogen and preventing this hormone from crossing the blood–brain barrier, thereby protecting the female brain from masculinization^89^.

Few studies described gestational age-dependent changes in the AF proteome, and a direct comparison between such studies and the results herein was not feasible. Therefore, we instead correlated the fold changes determined herein with our previously reported gestational age-dependent changes in the AF cell-free transcriptome. We observed a significant correlation (~ 0.6) among common differentially expressed genes and proteins in cell-free AF. Indeed, 83 genes or proteins were differentially abundant in the same direction between term and midtrimester samples in both the AF cell-free transcriptome and proteome, suggesting that they reflect shared processes related to fetal development and maternal adaptations.

Given the ultimate goal of developing non-invasive blood tests to predict, diagnose, and monitor pregnancy-related diseases, we sought to identify gestational age-modulated transcripts or proteins in the maternal circulation that mimic those reflected in the AF proteome. Consistent with this objective, we correlated the changes observed in the AF proteome to those in the maternal blood cellular transcriptome and maternal plasma proteome (that were assessed by the same platform). While no overall correlation was observed between the maternal plasma proteome and AF proteome, 14 genes coding for proteins significantly changing in abundance between the term and midtrimester AF samples also changed (in the same direction) with advancing gestation in the maternal blood. Of these 14 proteins, most are associated with neutrophil migration and degranulation, which aligns with a prior finding that maternal neutrophils can invade the amniotic cavity, especially in a late preterm or term gestation, and explains the observed association between the maternal blood transcriptome and AF proteome^75^. For example, LTF is an anti-bacterial, iron-chelating protein released by neutrophils^90^. The AF concentrations of this molecule were shown herein and previously to increase with gestation before decreasing during spontaneous labor at term^90^. Moreover, the AF LTF concentration dramatically increased in both term and preterm gestations that presented intra-amniotic infection^90^. LTF concentration was also higher in women who delivered prematurely after experiencing preterm labor than who delivered at term^91^. Maternal serum LTF levels are also inversely associated with fetal birth weight, indicating its potential as a biomarker for non-invasive fetal growth monitoring^92^. Similar to LTF, CRISP3 and BPI are stored and secreted from neutrophil granules and play a role in host defense^93^. Notably, whole-blood RNA sequencing showed over-expression of CRISP3 and BPI mRNAs among women with cervical insufficiency^94^. OLR1 is a multiligand C-type lectin receptor preferentially expressed in syncytiotrophoblasts, cytotrophoblasts, Hofbauer cells, and macrophages^58^. Upon receptor-ligand interaction, OLR1 can stimulate ROS production, NF‐κB signaling, apoptosis, and cell-mediated antigen cross-presentation^86^. Particularly, OLR1 was shown to play a role in the induction of overreaction and suppression of migration in neutrophils^95^. Therefore, our findings demonstrate consistent evidence that neutrophil-related immune processes are upregulated in the maternal circulation and in the amniotic cavity throughout gestation.

### Strengths and limitations

This study represents one of the most extensive characterizations of changes in the AF proteome during normal pregnancy. The AF concentrations of 1310 proteins were simultaneously assayed by utilizing a multiplex, affinity-based, proteomic-platform SOMAscan, which has been proven to possess high sensitivity and high specificity over a wide dynamic range. The study was sufficiently powered to detect changes in AF protein concentrations between the midtrimester and term samples as well as between the early and late midtrimester samples. Previous reports of gestational age-dependent changes in the AF cell-free transcriptome, maternal blood cellular transcriptome, and maternal plasma proteome within the same population allowed correlation of the findings across omics platforms and biological fluids. This information can potentially inform future study designs that target biomarkers of obstetrical disorders in less invasively collected fluids, such as the maternal peripheral blood. An additional strength of this study is that it provides data in a subset of women, mostly self-identified as African American, which could facilitate efforts to reduce disparity in pregnancy and neonatal outcomes.

The main limitation of this study is that amniocentesis is an invasive procedure; therefore, repeated sampling for each patient, which would be ideal for assessing temporal changes, was not feasible. In addition, the version of the SOMAscan assay used herein targets only the sub-proteome (1310 proteins) of AF. Close to 3000 AF proteins have thus far been identified in second-trimester AF; therefore, since the complete AF proteome was not examined, GO enrichment analysis may not provide a complete picture of the underlying physiology. Another limitation is that the SOMAscan assay provides fluorescence-based abundance instead of absolute concentrations, prohibiting direct comparisons of raw protein expression across batches and platforms. However, the primary objective of this study was to describe changes in protein expression between groups defined according to gestational age. Thus, the fold changes in protein expression determined herein may be compared across proteomic platforms, such as immunoassays or mass-spectrometry-based assays, without experimental bias. Further studies are required to determine whether our findings are applicable to women of other ethnicities.

### Conclusion

To our knowledge, this is the first study to apply an aptamer-based assay to profile the AF proteome and its modulation with gestational age. Overall, we demonstrated agreement with previously published cell-free RNA data regarding increased activity related to specific fetal organs, such as the brain, as well as a decrease in those related to gestational tissues, such as the placenta. Notably, this study suggests that the proportion of AF proteins modulated with advancing gestation is substantially greater than that of maternal plasma proteins. The normal gestation-associated AF proteomic modulation reported herein may serve as a reference for future studies aimed at discovering novel biomarkers for obstetrical diseases.

## Materials and methods

### Study design

Pregnant women who sought care at the Center for Advanced Obstetrical Care and Research of the Perinatology Research Branch, *Eunice Kennedy Shriver* National Institute of Child Health and Human Development (NICHD), National Institutes of Health, U.S. Department of Health and Human Services in the Detroit Medical Center and Wayne State University were enrolled in a prospective study. From this cohort, a retrospective cross-sectional study was designed to include 28 women: 15 women who underwent transabdominal amniocentesis during the midtrimester for genetic testing, and 13 women who underwent amniocentesis at term (not in labor, TNL) to assess fetal lung maturity (n = 10) or during cesarean delivery for research purposes (n = 3). We excluded women with the following complications from the study: intrauterine fetal demise, preeclampsia, eclampsia, HELLP (hemolysis, elevated liver enzymes, low platelet count) syndrome, chronic hypertension, gestational hypertension, gestational diabetes mellitus, pregestational diabetes, small-for-gestational-age neonate, sonographic short cervix, preterm labor, preterm prelabor rupture of the membranes, clinical chorioamnionitis, acute inflammatory lesions (stage 2 or higher maternal or fetal inflammatory response^96^), multiple gestation, fetal malformations, and genetic anomalies. The Institutional Review Boards of Wayne State University and NICHD approved the study protocols. All mothers provided informed written consent for the use of biological specimens and associated metadata for research prior to the collection of all samples. All methods were performed in accordance with relevant guidelines and regulations.

### Amniotic fluid samples

Amniotic fluid was withdrawn either transabdominally by a 22-gauge needle while monitoring with ultrasound or by direct aspiration through intact membranes during cesarean delivery. AF was collected in a capped, sterile syringe and immediately transported to the research laboratory.

Amniotic fluid contains increased amounts of fetal cells, hair, and vernix as the pregnancy advances^97^. Herein, all amniotic fluid samples were centrifuged at 1300 × g for 10 min at 4 °C, and the clear AF supernatant free of any particulate matter was stored at −80 °C, which was used for proteomics analysis^98–100^. Furthermore, samples contaminated by meconium or blood were not included in this study.

### Proteomics

The abundance of 1310 proteins in the amniotic fluid samples was determined with the SOMAmer (Slow Off-rate Modified Aptamers) platform by Somalogic, Inc. (Boulder, CO, USA), as previously described^32^. Briefly, three serial dilutions of AF samples were incubated with respective SOMAmer mixes pre-immobilized onto streptavidin-coated beads. Each dilution is targeted by a unique subset of SOMAmer reagents designed for a specific detection range, thereby allowing robust measurement across a wide dynamic range^101^. The non-specifically bound proteins and other matrix constituents were removed from the beads by washing. The NHS-biotin reagent was used to tag the proteins specifically bound to their cognate SOMAmer reagents. To prevent any non-specific interactions from re-forming, the beads were exposed to an anionic competitor solution. Pure cognate-SOMAmer complexes and unbound SOMAmer reagents were released from the beads by breaking the photo-cleavable linker (in SOMAmer reagents) through exposure to ultraviolet light. The photo-cleavage eluate released from the beads was then incubated with a second streptavidin-coated bead to capture the biotinylated proteins (and bound SOMAmer reagents). Any unbound SOMAmer reagents were removed during subsequent steps of washing. The bound SOMAmer reagents were separated from their cognate proteins under denaturing conditions and hybridized to custom DNA microarrays. The Cyanine-3 signal from the fluorophores (in SOMAmer reagents) was detected on microarrays to quantify protein abundance in relative fluorescence units. Standardization of the raw signal intensities included hybridization normalization to each sample to correct for hybridization variation within a run, median normalization across samples to remove other assay biases (e.g., variation in pipetting, reagent concentrations, and assay timing) within a run, and calibration normalization to adjust for plate-to-plate variation. As a final step, the standardized intensities were log_2_ transformed to improve normality. The log_2_ transformed protein abundance data is provided in Table S5.

### Data analysis

#### Demographics data analysis

The study participants’ clinical characteristics were summarized as the median and inter-quartile range for continuous variables and as proportions for categorical variables. To compare between gestational-age groups, the Welch’s t-test^102^ and Fisher’s exact test^103^ were used for continuous and categorical variables, respectively. A *p*-value < 0.05 was considered statistically significant.

#### Differential abundance analysis and validation

The abundance of 1310 proteins in the amniotic fluid was compared between samples collected during midtrimester and at TNL by fitting linear models implemented in the *limma*^104^ package. The linear models included, as covariates, variables whose distributions were significantly different between groups (fetal sex and maternal age). A minimum fold change of 1.5-fold and a false discovery rate^105^ adjusted *p*-value (q-value) < 0.1 were used to determine statistical significance. This threshold was chosen because SOMAscan is a microarray-based assay, and similar cutoff values have been validated extensively with the use of alternative techniques and independent samples in high-throughput microarray platform-based studies^106–109^. The results of differential expression analysis were summarized and visualized with volcano plots and heatmaps.

Amniotic fluid concentrations of two differentially abundant proteins (IL-6 and IL-8) were available in the Perinatology Research Branch database for a subset of samples used in this study. Such determinations were performed by specific immunoassays (Meso Scale Discovery, Rockville, MD, USA, and R&D Systems, Minneapolis, MN, USA), according to the manufacturers’ instructions. The available cytokine concentrations were used as confirmatory data for SOMAscan assay-derived relative abundance, and Spearman’s correlations were determined.

The gestational age-dependent changes in the AF proteome were also compared to previous reports of gestational age-dependent changes in the AF cell-free transcriptome^49^, maternal plasma proteome^32^, and maternal blood cellular transcriptome^50^. To assess agreement, we calculated the Spearman’s correlation between log_2_ fold changes with gestational age obtained herein and those previously reported for corresponding molecules (proteins/genes) significant in each pair of studies.

#### Clustering differentially abundant proteins

We used WGCNA^110^ to identify patterns of change across gestation among the proteins differentially expressed between the early and late midtrimesters or between combined midtrimester and term groups. The following parameters were used: networkType = signed, corType = bicor, and power = 22. The remaining parameters were set to default. WGCNA summarizes clusters/modules of interconnected proteins with module eigenvalues that can be used to identify intra-modular hub molecules^110,111^. We used locally estimated scatterplot smoothing (LOESS) regression to plot the gestational age-dependent expression of four intra-modular hub proteins from each module.

#### Gene ontology enrichment analysis

All proteins were mapped to Entrez gene identifiers^70^ per the manufacturer’s provided annotation. A hypergeometric test implemented in the *GOstats* package^112^ was used to identify significantly enriched GO^113^ biological processes, molecular functions, and cellular components among genes coding for the differentially regulated proteins. The genes corresponding to the 1310 proteins evaluated on the SOMAscan assay were used as the background list. The analysis was restricted to GO terms with at least three hits. An enrichment q-value < 0.05 was considered statistically significant.

#### Tissue-specific expression

To determine the tissues or cell types associated with the observed protein-abundance changes in advancing gestation, we defined tissue/cell type-specific genes as those with a median expression 30 times higher in a given tissue than all other tissues in the GNF Gene Expression Atlas^51^. The log_2_ transformed expression values for each protein coded by these genes were standardized by subtracting the mean and dividing by the standard deviation calculated from the reference study group (midtrimester). The standardized values for a maximum of the top 20 genes preferentially expressed in a tissue and measured on the SOMAscan platform were averaged to obtain the tissue-specific Z scores^49,76^. Z-scores were compared between groups with the Wilcoxon rank-sum test. A q-value of < 0.05 was considered significant.

## Supplementary Information

Supplementary Information 1.

Supplementary Information 2.

Supplementary Information 3.

Supplementary Information 4.

Supplementary Information 5.

Supplementary Information 6.
