# Supplementary material for: The amniotic fluid proteome changes with gestational age in normal pregnancy: a cross-sectional study
Source: Sci Rep. 2022 Jan 12;12:601. doi: 10.1038/s41598-021-04050-9 (PMC8755742; doi:10.1038/s41598-021-04050-9)
Supplement: Supplementary file 1 — Supplementary Information 1. [file 41598_2021_4050_MOESM1_ESM.doc]

**Supplementary Table Legends**

**Table S1: Proteins significantly changing in abundance between** **amniotic fluid samples collected at term not in labor (TNL) and during midtrimester.** The table includes the gene symbol, protein name, ENTREZ database identifier, log_2_ fold change, and adjusted p-value (q-value).

**Table S2:** **Gene Ontology enrichment analysis of genes coding for amniotic fluid proteins increased in abundance at term not in labor (TNL) compared to midtrimester.** Count: number of differentially expressed genes associated with the Gene Ontology term. Size: total number of genes related to the Gene Ontology term; odds ratio of enrichment based on a Fisher’s exact test; q-value: adjusted p-value.

**Table S3: Gene Ontology enrichment analysis of genes coding for amniotic fluid proteins decreased in abundance at term not in labor (TNL) compared to midtrimester.** Count: number of differentially expressed genes associated with the Gene Ontology term. Size: total number of genes related to the Gene Ontology term; odds ratio of enrichment based on a Fisher’s exact test; q-value: adjusted p-value.

**Table S4: Proteins significantly changing in abundance between amniotic fluid samples collected during the early (16.4–21.0 weeks) and late (21.1 -24 weeks) midtrimesters.** The table includes the gene symbol, protein name, ENTREZ database identifier, log_2_ fold change, and adjusted p-value (q-value).

**Table S5**: **Proteomics data used in the analyses presented in this study**. The table includes the log_2_ transformed protein abundance data for each sample (rows) and each of the 1310 proteins. ID: anonymized identifier indicator of the mother, Age: Maternal age, Sex: Fetal sex, Group: Midtrimmester or term not in labor (TNL).
